# Increased resistance to a generalist herbivore in a salinity-stressed non-halophytic plant

**DOI:** 10.1093/aobpla/plw028

**Published:** 2016-07-11

**Authors:** Sylvie Renault, Scott Wolfe, John Markham, Germán Avila-Sakar

**Affiliations:** ^1^Department of Biological Sciences, University of Manitoba, Winnipeg, Manitoba R3T 2N2, Canada; ^2^Department of Biology, University of Winnipeg, 599 Portage Ave, Winnipeg, Manitoba R3B 2G3, Canada

**Keywords:** Abiotic stress, cross-talk, glycophyte, insect herbivore, resistance, tolerance

## Abstract

Plants often grow under the combined stress of several factors. Salinity and herbivory, separately, can severely hinder plant growth and reproduction, but the combined effects of both factors are still not clearly understood. Salinity is known to reduce plant tissue nitrogen content and growth rates. Since herbivores prefer tissues with high N content, and biochemical pathways leading to resistance are commonly elicited by salt-stress, we hypothesized that plants growing in saline conditions would have enhanced resistance against herbivores. The non-halophyte, *Brassica juncea*, and the generalist herbivore *Trichoplusia ni* were used to test the prediction that plants subjected to salinity stress would be both more resistant and more tolerant to herbivory than those growing without salt stress. Plants were grown under different NaCl levels, and either exposed to herbivores and followed by removal of half of their leaves, or left intact. Plants were left to grow and reproduce until senescence. Tissue quality was assessed, seeds were counted and biomass of different organs measured. Plants exposed to salinity grew less, had reduced tissue nitrogen, protein and chlorophyll content, although proline levels increased. Specific leaf area, leaf water content, transpiration and root:shoot ratio remained unaffected. Plants growing under saline condition had greater constitutive resistance than unstressed plants. However, induced resistance and tolerance were not affected by salinity. These results support the hypothesis that plants growing under salt-stress are better defended against herbivores, although in *B. juncea* this may be mostly through resistance, and less through tolerance.

## Abstract (precis)

During their life, plants face multiple stresses. However, studies on one stress factor have typically neglected possible interactions with other factors. We demonstrated that salt stress in Indian mustard (a plant species not adapted to salinity) lessens the effect of herbivory on plant mass, and increases the plants' constitutive resistance to herbivores. Changes in the plants associated with increased salt that help to explain the mitigation of herbivore effects include decreased protein and macronutrient content. Plants exposed to herbivore damage were also less negatively affected by salt exposure, possibly due to their ability to maintain higher levels of transpiration.

## Introduction

Around 350 million hectares of land across the globe are affected by increasing salt levels (Rengasamy 2006). Salinization has increased as a result of the redistribution of salts in soil during the conversion of wetlands or forests into agricultural land. Although salinization of soil is most common in arid and semi-arid regions, it has been reported in almost all climatic regions (Mittal *et al.* 2012). These increased salt levels have detrimental effects on plant growth and productivity and have left extensive areas of natural and agricultural land degraded (Orcutt and Nilsen 2000). Halophytes are plants naturally adapted to growing in saline areas, but non-halophytes, which include many of our crops, show a wide range of responses to salinity, from low to relatively high tolerance, as measured by seed germination, survival, growth rate, reproduction and physiological processes such as water uptake, transpiration and accumulation of solutes and certain ions (Greenway and Munns 1980; Sairam and Tyagi 2004).

Direct effects of salinity include reduced water uptake (osmotic stress) and increased uptake of ions (Na^+ ^and Cl^−^) that may inhibit enzymatic activity (ionic toxicity) and may also result in nutrient imbalance leading to nutrient deficiency (Munns and Tester 2008; Deinlein *et al.* 2014). These direct effects of salinity may hinder the growth of both vegetative and reproductive structures, and also obstruct the ability of plants to defend themselves from herbivores and other natural enemies. Furthermore, the alteration of the photosynthetic electron transport system caused by salinity can lead to the production of reactive oxygen species (Munns and Tester 2008), which may further damage the plants by causing oxidative damage to membranes, proteins and nucleic acids.

Plant growth is usually affected by both biotic and abiotic environmental factors (Shao *et al.* 2007). In most cases, biotic stressors like herbivory have not been considered when studying salt stress (cf. Griffith and Anderson 2013), even though plants growing in saline environments are not immune to herbivore attack.

To predict the effects of salt stress on plant defence against herbivores, one must consider how salinity affects not only tissue quality, but also the physiological processes and biochemical pathways underlying growth, reproduction and the production of physical and chemical resistance traits (trichomes, wax, lignin, secondary metabolites, etc.), all of which ultimately influence plant resistance and tolerance to herbivory (Karban and Myers 1989; Wu and Baldwin 2010). Given that, in the short term, salinity causes osmotic stress in plants, which induces biochemical responses that interact with the response of plants to herbivory (Wang *et al.* 2001; Kessler and Baldwin 2002; Thaler and Bostock 2004; Rejeb *et al.* 2014; Dar *et al.* 2015) and causes a decrease in tissue water content (Deinlein *et al.* 2014), one would predict that herbivores would initially avoid salt-stressed plants, thus resulting in increased resistance under salinity. In the longer term salt-stressed plants could also suffer a decrease in tissue nitrogen content (mostly in the loss of chlorophyll and rubisco) (Grattan and Grieve 1999; Mittal *et al.* 2012). Given the preference of herbivores for nitrogen-rich tissues, such decrease in nitrogen content would result in greater resistance (Herms 2002). However, given that herbivores require sodium in their diets, as sodium accumulates in plant tissues, they should become more attractive to herbivores, thus resulting in decreased resistance under salinity (Pilon-Smits *et al.* 2009). Integrating both responses, the actual effect of salinity on plant resistance against herbivores would be determined by the balance between the changes in sodium and nitrogen content, and the relative need of each element in an herbivore’s diet. Given that, usually, insects need more nitrogen than sodium in their diets (Joern *et al.* 2012), we predict that changes in plant nitrogen would drive herbivore preference, thus resulting in greater resistance of salt-stressed plants. Moreover, tissue concentration of secondary metabolites—including glucosinolates, the main secondary metabolites in the mustard family—generally increases in response to salinity stress (Sabra *et al.* 2012; Martínez-Ballesta *et al.* 2013; Rodziewicz *et al.* 2014; Garg *et al.* 2015; Forieri *et al.* 2016). Thus, based on the biochemical signal cascade elicited by salinity, we would also expect greater resistance in salt-stressed plants.

As for tolerance, since salt-stress slows down plant growth (Deinlein *et al.* 2014 and references therein), we would expect greater tolerance for plants growing under salinity (Hilbert *et al.* 1981; Avila-Sakar and Laarakker 2011). While the prediction of greater tolerance in slower growing plants might not seem intuitive, it stems from the fact that tolerance is defined, explicitly or not, in reference to an undamaged control (Strauss and Agrawal 1999). Hence, all else being equal, the difference in biomass produced (correlated to reproductive output) between undamaged plants and those subjected to a particular amount of damage will be smaller when undamaged control plants grow more slowly (Hochwender *et al.* 2000). Such a prediction might apply better to non-stress-tolerant plants since, the ability of stress-tolerant plants to tolerate abiotic stresses such as salinity is linked to their inherently low growth rates, which in turn are associated to a high degree of herbivore resistance and a low level of herbivore tolerance (Grime 1977). In contrast, non-stress-tolerant plants have higher potential growth rates, and putatively less resistance but more tolerance to herbivory.

In halophytes, resistance to herbivory has been found to decrease (Gonçalves-Alvim *et al.* 2001; Nabity *et al.* 2006), increase (Hemminga *et al.* 1987; Hemminga and van Soelen 1988), or not change (Hemminga and Van Soelen 1992) in response to salt stress, and in some plants the result varies with herbivore species (Moon and Stiling 2002*a*, *b*). As for non-halophytes, susceptibility to herbivory was unaffected in trees damaged by de-icing salts in an urban environment (Munck *et al.* 2010). In contrast, leafminer density dropped (suggesting increased resistance), for *Iris hexagona* grown under saline conditions (Schile and Mopper 2006). Clearly, the effect of salinity on resistance is unresolved, and to date, studies of non-halophytic plants are lacking. Thus, the main objective of this study was to determine whether non-halophyte plants growing under salt-stress were more resistant and tolerant against herbivores than plants growing without the stress of salinity.

## Methods

### Plant material

Indian mustard, *Brassica juncea* (Brassicaceae) was selected for this study for its moderate tolerance to salinity (Purty *et al.* 2008). It is an amphidiploid hybrid of *Brassica campestris* and *Brassica nigra* that is able to withstand environmental stress better than its diploid counterparts (Ashraf and McNeilly 2004). *Trichoplusia ni* (cabbage looper) was chosen as the herbivore to test resistance and tolerance to herbivory in *B. juncea* because, as a generalist, leaf-chewing herbivore, it is a common pest in many crops, including those in the mustard family (Cameron *et al.* 2007). *Brassica juncea* produces seeds through autonomous self-fertilization (Yashiro *et al.* 2001).

### Experimental design and plant growth

Our approach was to use a hydroponic system to expose plants to salt treatments. We then measured the growth, physiological responses and herbivore resistance of a subset of plants. The remaining plants were exposed to herbivores and had half of their leaves removed, or left as controls, to examine the effect of the salt treatment on induced herbivore resistance and tolerance. These plants were grown to maturity and their fitness (seed yield) measured.

*Brassica juncea* var. cutlass seeds were germinated in Petri plates for one week under fluorescent lights (125–150 μmol s^−^^1^ m^−^^2^). After one week, 5 mL of half-strength modified Hoagland’s nutrient solution (Sabra *et al.* 2012) was added to each Petri plate and the seedlings were left to grow for another week. Six randomly chosen seedlings were then transferred to each of 15 10-L plastic containers (a total of 90 plants) filled with half-strength modified Hoagland’s solution that was kept aerated using an aquarium pump (Renault *et al.* 2001). Plants were grown at 25 °C, under a 14:10 h light:dark photoperiod for 2 weeks to allow for their acclimation to hydroponic conditions. When plants were 4 weeks old, each container was randomly assigned to one of three salinity treatments consisting of 0, 50 or 100 mM NaCl solutions prepared in half-strength modified Hoagland’s nutrient solution. Thus, each salinity treatment was replicated five times. To avoid osmotic shock, seedlings in the 100 mM NaCl treatment were exposed to 50 mM NaCl for 6 h prior to increasing the concentration to 100 mM. Conductivity and water levels were monitored daily to keep the salt and nutrient concentrations constant (7.15 dS m^−^^1^ for 50 mM NaCl and 12.50 dS m^−^^1^ for 100 mM). The hydroponic solutions were replaced weekly to avoid nutrient deficiency.

After 2 weeks in their salinity treatments (at an age of 6 weeks), two randomly selected plants from each treatment (a total of 30 plants) were harvested and used to ascertain the effects of salinity on tissue quality and plant growth. The harvested plants were washed three times with distilled water, and the fresh weights of roots, stems and leaves were determined. Leaf area of fresh leaves was measured using a leaf area meter (LI-COR, Nebraska, USA). Plant parts were lyophylized to obtain their dry weights. Leaf tissue quality was assessed in terms of specific leaf area (SLA, calculated per plant as: total leaf area of plant/total leaf dry weight), chlorophyll, crude protein, proline and water content. Leaf water content was determined from the fresh and dry weights of four leaf disks (0.6 cm^2^) from each plant. Leaf chlorophyll content was determined by spectrophotometry (650 and 665 nm) of three methanol washes from similar leaf disks (Renault *et al.*, 2001). To determine the crude protein content of the leaves, frozen samples (0.5 g) were ground in liquid nitrogen. Proteins were extracted with 25 ml of cold phosphate buffer (0.05 M; pH 7.0) containing 1 mM ethylenediaminetetraacetic acid (EDTA) and 1 mM L-ascorbic acid, along with 1% polyvinylpyrrolidone (PVP) (Jones *et al.* 1989). The homogenate was kept on ice for 20 min. After extraction the homogenate was centrifuged at 4 °C for 20 min at 15 000 *g*. The supernatant (200 μl) was mixed with 5 ml of Comassie Brilliant Blue G-250 reagent and the absorbance was read at 595 nm (Bradford 1976). Bovine serum albumin was used as a standard. To determine leaf proline content, a modified Bates method (Bates *et al.* 1973) was used. Proline was extracted with 10 ml of sulphosalicylic acid (3%) for 30 min and centrifuged for 5 min at 4900 *g* from frozen leaf tissues (0.5 g) previously ground in liquid nitrogen. The supernatant (1 ml) was incubated with 2 ml of a 60% acetic acid and 1% ninhydrin reagent for 1 h at 100 °C. This solution was then cooled on ice, 3 ml of toluene were added and the 2 phases rigorously mixed. After separation of the phases, the organic phase was isolated and its absorbance read at 520 nm. Proline content was determined from a standard curve prepared using standard L-Proline (Sigma-Aldrich). Lyophylized ground tissues were used to determine the nutrient and Na content of the leaves. Samples were analyzed with a CHNOS elemental analyser ‘vario Micro’ (Elementar, Hanau, Germany).

Of the remaining four plants from each replicate of each salt treatment, two were randomly assigned to an herbivory treatment and also used to obtain leaf disks for bioassays to assess constitutive and induced resistance to herbivory. The other two plants were kept without herbivory. For the herbivory treatment, four *T. ni* larvae were placed on each plant and allowed to feed on its leaves for 4 h. Larvae consumed roughly one third of the leaf area on each plant. Larvae were constrained to feed on the leaves only, and kept away from the flowers. After the larval feeding, we also simulated herbivory on these plants by manually removing half the leaves from one side of the plant.

Two weeks after the herbivory treatments were applied (at an age of 8 weeks and a size too large to be kept in hydroponic growth), all plants were transferred to pots with a 1:2:1 (V:V:V) mix of sand, peat and perlite containing 0, 50 and 100 mM of NaCl. The soil moisture levels were examined daily and distilled water was added accordingly to keep the soil moist. Two weeks after being transplanted (4 weeks after the application of herbivory treatments) transpiration and stomatal conductance was measured on undamaged leaves on all plants. As plants senesced, all mature fruits were collected and air-dried at room temperature; their seeds were counted and weighed. Senescent (dry) plants were harvested and separated into roots, stems and leaves, oven-dried at 62 °C for 3 days and weighed.

Constitutive and induced resistance of plants to herbivores were assessed by means of bioassays using *Trichoplusia ni*. Eggs of *T. ni* were obtained from the Canadian Forest Service (Insect Production Services) and reared on the McMurran artificial diet from the same supplier at 21 °C until they reached the late third or early fourth instar (Tucker and Avila-Sakar 2010). Choice assays were conducted using larvae that had been starved for 20 h. Larvae were individually placed in Petri plates and presented with three 0.6 cm^2^ leaf disks, each freshly cut from mature leaves of a plant grown in one of the three salinity treatments (Hoque and Avila-Sakar 2015; Kornelsen and Avila-Sakar 2015). The disk area remaining after 40 min was measured with a portable leaf area analyzer, and used to estimate resistance as:

$$R=A_{f}/A_{i}$$

where *R* is resistance, *A*_i_ is the initial area of the leaf disk and *A*_f_ is the disk area remaining after exposure to the larva. Two sets of disks per plant were tested, and the estimates of resistance obtained were then averaged for each plant. For constitutive resistance, leaf disks were cut from plants assigned to the herbivory treatment before larvae were placed on plants. For induced resistance, leaf disks were obtained one day after larvae had fed on plants. We estimated tolerance to herbivory as the difference between the mean life-time seed production of damaged and undamaged plants within a replicate of salinity level: *Delta-seeds = S*_d_ *− S*_u_. In this manner, a positive value indicates over-compensation, a value of zero indicates exact compensation and a negative value indicates under-compensation (Strauss and Agrawal 1999).

### Data analysis

Data from the plants within a replicate (grown in the same container) were combined to avoid pseudoreplication. The effects of salinity on plant performance before the herbivory treatment, and resistance to herbivores after herbivory treatments were analyzed using least squares regression, with the data transformed when variances between treatments were not homogeneous. For the plant measurements made after exposure to herbivores, data were analyzed using an ANCOVA model with herbivore treatment as a categorical variable and salt content as a covariate.

## Results

After 2 weeks of growth under saline conditions, the total biomass of plants exposed to 50 and 100 mM NaCl was, respectively, 24 and 35% less than that of plants in the no-salt treatment (Table 1). These reductions were driven by decreases in leaf, but not root or stem mass.

**Table 1.** Growth and tissue quality of *Brassica juncea* exposed to 0, 50 and 100 mM NaCl for 2 weeks. Values represent means ± SE (*n* = 5). *P* values are from linear regressions. *Indicates analysis was performed on log transformed data.

	NaCl			*P* value
	0 mM	50 mM	100 mM	

Total mass (mg)*	1698 ± 267	1293 + 159	1101 + 80	0.035
Root mass*	348 ± 40	289 ± 20	257 ± 22	0.119
Stem mass*	189 ± 18	178 ± 28	142 ± 27	0.286
Leaf mass*	1162 ± 137	826 ± 84	701 ± 65	0.025
Root:shoot	0.26 ± 0.02	0.30 ± 0.02	0.31 ± 0.02	0.129
SLA	464 ± 29	518 ± 39	483 ± 32	0.727
Leaf water content (%)	93.5 ± 0.1	94.0 ± 0.1	94.3 ± 0.1	0.165
Leaf protein (mg g^−1^)*	4.00 ± 0.98	3.37 ± 0.55	1.78 ± 0.28	0.030
Proline (µmol g^−1^)*	0.17 ± 0.04	0.44 ± 0.17	2.02 ± 0.63	0.001
Chlorophyll (mg g^−1^)	1.50 ± 0.09	1.34 ± 0.04	1.09 ± 0.06	0.001
Transpiration (mmol H_2_O m^−2^ s^−1^)	9.62 ± 0.85	9.38 ± 0.78	8.54 ± 1.05	0.576

**Figure 1.**
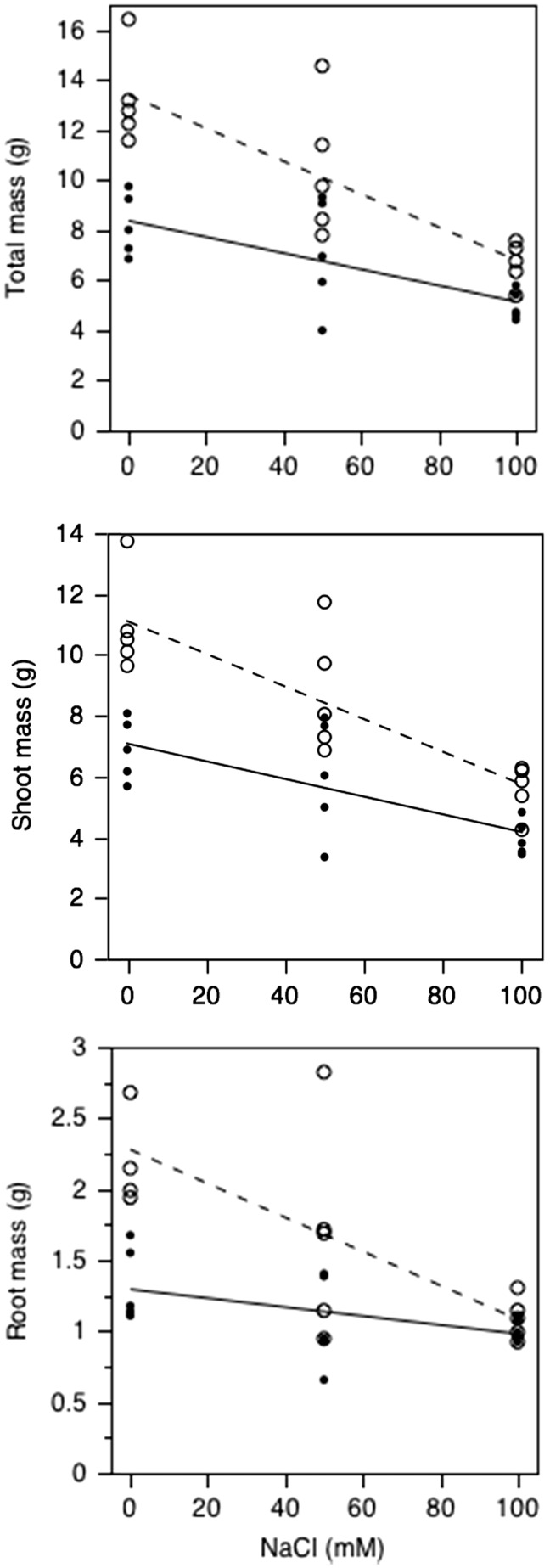
Effect of salt and herbivory on total, shoot and root final biomass of herbivore-damaged (closed symbols) and undamaged (open symbols) *Brassica juncea* plants. Each point is the mean of two plants harvested from a hydroponic container. The dashed and solid lines are the least squares lines from the ANCOVA model for the undamaged and damaged plants, respectively.

Final plant biomass was also negatively affected by salinity (Fig. 1, *F*_1,26 _=_ _41.6, *P* < 0.0001 for the effect of salinity). Plants in the 50 and 100 mM NaCl were 18.7 and 45.8% smaller, respectively, than those in the no-salt treatment. These trends, were less pronounced for plants subjected to herbivory, i.e. while herbivory had an negative effect on final plant biomass (*F*_1,26 _=_ _29.3, *P* < 0.0001 for the herbivory effect), this negative effect decreased as salinity increased (*F*_1,26 _=_ _4.8, *P* = 0.046 for the interaction between NaCl and herbivory treatment). This change in final biomass between herbivore and non-herbivore exposed plants was driven mainly by changes in root biomass, as there was no interaction between salinity and herbivory on shoot mass but there was for root mass (*F*_1,26 _=_ _7.2, *P* = 0.013). This shows that the reduced mass of the herbivore-exposed plants was not just a result of removal of leaf tissue.

The 2-week exposure to salt also resulted in a significant decrease in the crude protein content of the leaves, with plants exposed to 100 mM NaCl having less than half the protein content of the control plants, as well as a significant decrease in leaf chlorophyll content. However, salt-exposed plants had large increases in proline levels with 50 and 100 mM NaCl plants having, respectively, 2.6- and 11.9-fold increases compared to control plants. The salt treatments had no effect on root:shoot ratio, SLA, leaf water content or transpiration. Plants growing under salinity suffered significant decreases in tissue content of most macronutrients (N, K, Mg, Ca but not P; Table 2). However, the level of Na and some micronutrients (Cu, Mn and Zn but not Fe or B) increased. The largest change was in the Na content which increased 360 and 650 times in plants in the 50 and 100 mM NaCl treatments, compared to those grown in 0 mM NaCl.

**Table 2.** Macro- and micro-nutrient and Na content of *Brassica juncea* leaves exposed to 0, 50 and 100 NaCl mM for 2 weeks. Values represent means ± SE (n = 5).

Element	NaCl			*P*
	0 mM	50 mM	100 mM	

N (%)	7.0 ± 0.9	6.4 ± 0.6	5.3 ± 0.2	0.0013
P (%)	0.7 ± 0.2	0.8 ± 0.1	0.7 ± 0.1	0.929
K (%)	6.6 ± 1.2	2.9 ± 0.8	1.6 ± 0.2	<0.0001
Mg (%)	0.5 ± 0.1	0.4 ± 0.1	0.31 ± 0.04	0.0003
Ca (%)	2.9 ± 0.12	2.4 ± 0.7	1.7 ± 0.1	0.0005
Na (%)	0.012 ± 0.008	4.3 + 0.2	7.8 + 0.1	<0.0001
B (µg g^−1^)	58 ± 2	58 ± 8	52 ± 4	0.118
Cu (µg g^−1^)	8 ± 3	11 ± 3	15 ± 7	0.025
Fe (µg g^−1^)	62 ± 5	64 ± 8	67 ± 6	0.294
Mn (µg g^−1^)	55 ± 14	70 ± 18	92 ± 12	0.0013
Zn (µg g^−1^)	83 ± 27	111 ± 36	148 ± 27	0.0038

**Figure 2.**
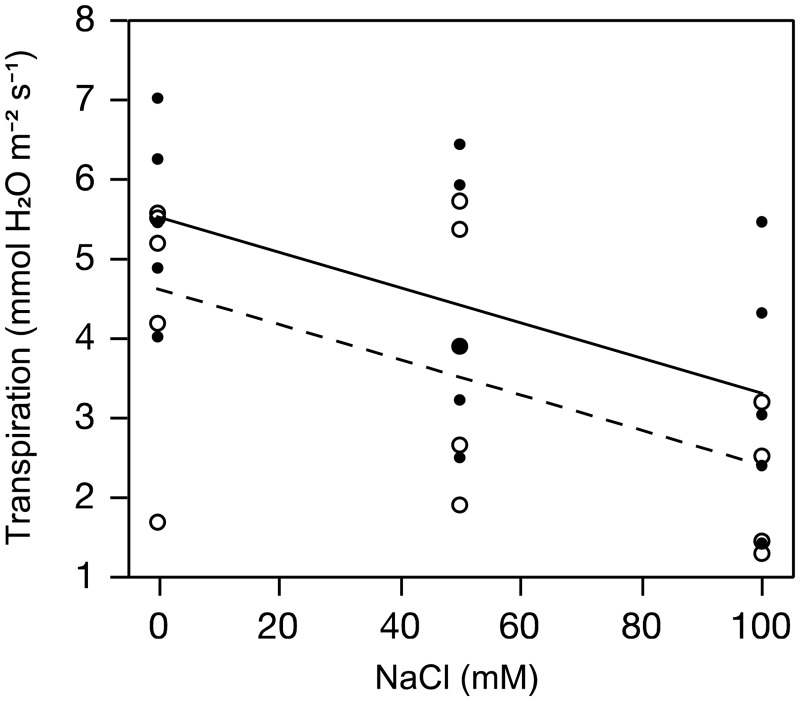
Transpiration rates of *Brassica juncea* leaves 4 weeks after herbivory treatments. Each point is the mean of the transpiration measured on the leaves of two plants harvested from a hydroponic container. The dashed and solid lines are the least squares fitted lines from the ANCOVA model for the undamaged (open symbols) and damaged (closed symbols) plants, respectively.

Four weeks following exposure to herbivores and simulated herbivory transpiration and stomatal conductance decreased as salinity increased (*F*_1,26 _=_ _12.6, *P* = 0.0014 and *F*_1,26 _=_ _13.2, *P* = 0.0012 for the salinity effect on transpiration and stomatal conductance, respectively; Fig. 2). The same trends were found in plants subjected to herbivory, except that they tended to have greater transpiration (36%) and stomatal conductance (33%) than undamaged plants (*F*_1,26 _=3.15, *P*= 0.087 and *F*_1,26 _=_ _3.8, *P* = 0.062 for the herbivory effect). There was no interaction between salinity and herbivory on transpiration or stomatal conductance (*F*_1,26 _=_ _0.001, *P* = 0.974 and *F*_1,26 _=_ _0.003, *P *= 0.954).

### Resistance and tolerance to herbivory

**Figure 3.**
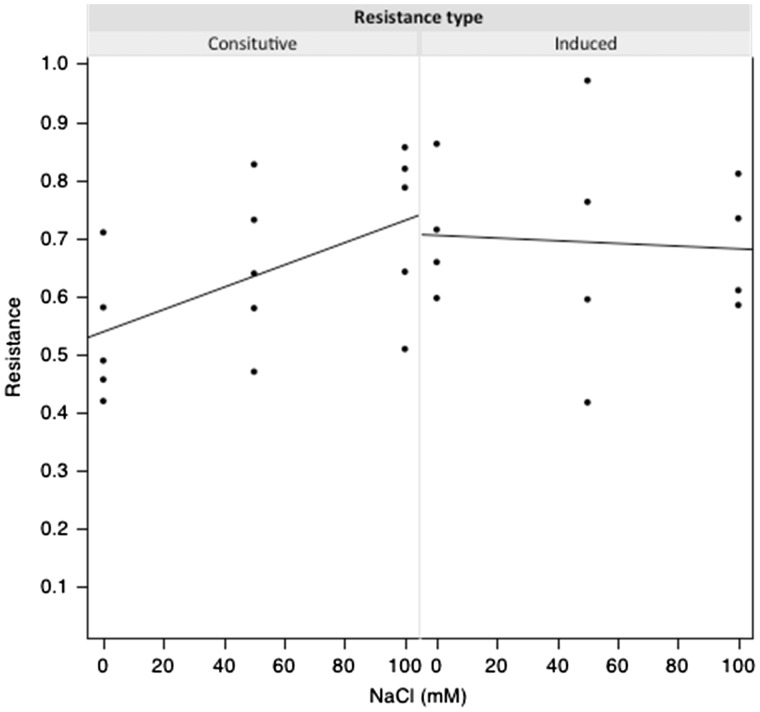
Constitutive and induced resistance of *Brassica juncea* plants exposed to different levels of NaCl in a leaf disk choice assay. Lines are least squares fits.

**Figure 4.**
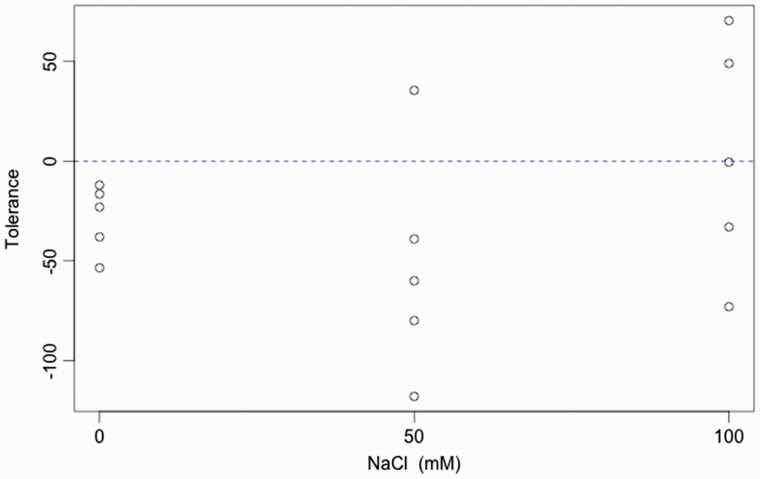
Tolerance to herbivory of *Brassica juncea* plants grown at different levels of NaCl. Tolerance was measured as the difference between the mean seed production of two individuals subjected to removal of 50% leaf area and that of two undamaged individuals per replicate (*n* = 5 per salinity level; see text for details).

Constitutive resistance increased with salinity (*F*_1,13 _=_ _5.559, *P* = 0.0347; Fig. 3). Plants grown at 100 mM NaCl were 36% more resistant than those in 0 mM NaCl. Interestingly, induced resistance levels did not vary with salinity level (*F*_1,13 _=_ _0.045, *P* =0.8370) but were similar to the mean constitutive resistance of plants grown at 100 Mm NaCl. We did not find a statistically significant effect of salinity on the tolerance of *B. juncea* to herbivory by *T. ni*, (Kruskal–Wallis chi-squared = 3.44, d.f. = 2, *P* = 0.1791; Fig. 4). We tested also a quadratic model, but it did not fit the data either (Kruskal–Wallis chi-squared = 2.94, d.f. = 1, *P* = 0.0864. We also observed that variability in the response to damage clearly increased in both the 50 and 100 mM NaCl treatments relative to the no-salt treatment. Herbivory had a detrimental effect on seed production of plants in the no-salt treatment (*Delta-seeds* value significantly less than zero; one-tailed Wilcoxon Signed Rank test, *V *= 0, *P* = 0.0312; Fig. 4), but such detrimental effect of herbivory was not evident at 50 or 100 mM NaCl.

## Discussion

Our results confirm a moderate tolerance to salinity in the variety of *B. juncea* selected for this study, as reported previously in most *Brassica* species (Purty *et al.* 2008). Although the salt-treated seedlings were able to maintain their transpiration rate and leaf water content, with no visible injury during the first 2 weeks of exposure to the salt treatments. After 4 weeks, the transpiration rate had decreased in inverse proportion to the salt concentration at which the plants were growing. The large proline leaf tissue content increase observed in the highest salt concentration could have contributed towards maintaining osmotic balance. Proline has been shown to accumulate in many plants in response to abiotic and biotic stresses, where it plays a protective role (Szabados and Savouré 2009). In addition to its role as a compatible osmolyte, proline can contribute to stress tolerance in a variety of ways including antioxidant function, protein protection and synthesis (as chaperone), and as a signalling molecule (Szabados and Savouré 2009 and references therein).

Our study shows that leaf tissue quality is affected by salinity. In addition to the decrease in biomass, proteins and chlorophylls of salt-stressed leaves also decreased—changes often attributed to the ionic stress caused by Na accumulation in tissues (Munns and Tester 2008; Mittal *et al.* 2012). A severe decrease in K was also observed in parallel to a drastic increase in Na. These changes in ion balance resulted in a high Na/K ratio, potentially toxic for plant cell metabolism. Decreases in other elements such as Ca and Mg were less pronounced. These changes have been attributed mainly to competition between ions during uptake at the roots. Na can compete with K, Mg and Ca for plasma membrane transporters (Zhu 2003; Hu and Schmidhalder 2005). The decrease in N content of leaf tissues, previously attributed to competition with Cl in salt-stressed plants (Hu and Schmidhalder 2005) further changed the nutritional quality of the salt-treated leaves. The higher level of micronutrients (Cu, Mn and Zn) in salt treated leaves, although present in relatively small amounts, may have also modified the nutritional value of the *B. juncea* leaves. These changes in the chemical composition of leaves will affect the food quality for the insect herbivores who usually require only trace amounts of sodium (Martel 1998).

Abiotic stress can increase plant susceptibility to attack by pathogens and herbivores (Herms 2002; Bostock 2005). Abiotically stressed plants may become less resistant to herbivory if their tissues become more nutritious (e.g. having a better balance of nitrogen, carbohydrates and minerals) to insects in response to stress (Mattson and Haack 1987). However, stress may also cause plant tissues to become less nutritious (Inbar *et al.* 2001). In our experiment, plants grown under salt-stress had increased levels of proline, a known insect feeding stimulant (Mattson and Haack 1987) and also of Na. However, our plants also suffered a decrease in total protein and N with increased salinity. Since constitutive resistance increased with salinity (larvae consumed less area from leaves exposed to increasing levels of NaCl), it seems that, as predicted, the reduction in available N was more important than the increase in either proline or Na for *T. ni* preference. Similarly, salt-treated leaves of *Solidago altissima* were less preferred by *Trirhabda borealis* larvae (Martel 1998).

In our experiment, induced resistance did not change in response to salinity but was consistently as high as the constitutive resistance achieved under the highest salt level. This suggests that salinity induces changes in tissue quality similar to those brought about by the feeding of *T. ni* larvae feeding on foliar tissue for 4 h. Plants have a complex set of responses to both biotic and abiotic stress that can potentially interfere with one another. Two plant growth regulators, ABA and jasmonic acid (JA) are known to play a key role in these responses as signal molecules (Thaler and Bostock 2004). ABA is produced in response to salinity and drought (Chaves *et al.* 2009). However, ABA also increases in response to wounding (Peña-Cortés and Willmitzer 1995) and its role as regulator of induced resistance to herbivory was reported in *Arabidopsis* (Vos *et al.* 2013). The second signalling compound, jasmonic acid is typically produced in response to herbivory to elicit defence mechanisms like the synthesis of proteinase inhibitors and enzymes involved in the production of secondary compounds (Wang *et al.* 2001; Bari and Jones 2009). However, salinity can also regulate the biosynthesis of jasmonic acid and induce the production of JA-responsive proteins (Moons *et al.* 1997). A previous study on the interaction between salinity and herbivory reported a similar increase in JA (Wang *et al.* 2001). Changes in these plant growth regulators could have contributed to the observed changes in resistance to herbivory. The crosstalk between these signalling compounds can be quite complex and constitutes a topic of intensive research (Fujita *et al.* 2006; Rejeb *et al.* 2014).

We did not find a significant effect of salinity on tolerance to herbivory. While plants in the no-salt treatment undercompensated, plants grown under saline conditions neither under- nor over-compensated. However, we should be cautious about interpreting this result as evidence of exact compensation under salinity because our lack of ability to detect a significant change in seed production between damaged and undamaged plants was due to a large variation in seed production among plants grown at 50 and 100 mM NaCl. In this regard, it is important to investigate the mechanisms by which salt-stress stimulated seed production in some plants, but reduced it in others. One possible mechanism for maintaining seed production after herbivore damage may be related to resource translocation from roots (Korpita *et al.* 2013; Nalam *et al.* 2013; Hoque and Avila-Sakar 2015; Kornelsen and Avila-Sakar 2015 ). We found that root mass was less affected by herbivory when the plants were exposed to salt. If these plants were able to translocate carbon and nitrogen into seeds they could mitigate the effect of lost leaf tissue on seed production. Moreover, since salt-stressed plants grew less than non-stressed plants in the same period, these results are consistent with the prediction that slower growing plants would have greater compensatory ability than those with higher growth rates (Hilbert *et al.* 1981, Avila-Sakar and Laarakker 2011), and with findings in *Arabidopsis* and *Asclepias* (Hochwender *et al.* 2000; Barto and Cipollini 2005; Tucker and Avila-Sakar 2010).

## Conclusions

Our results show that resistance against herbivores is enhanced by salinity stress. While Na and proline foliar tissue content increased in response to salinity, N content decreased; and herbivores preferred to feed on leaf tissue from plants grown without salt stress rather than that of salt-stressed plants. This suggests herbivore preference is more strongly affected by available N than by Na or proline.

Tolerance of herbivory was more variable among plants growing under salinity, and salt-stressed plants generally achieved better compensatory growth of roots. While further investigation into the effects of salinity on tolerance to herbivory is needed, so far, it would seem of little use to eliminate insect pests from crops growing in saline soils or plants used for restoration of high salinity sites. This being one of the few studies of the effects of salinity on defence against herbivores on non-halophytes, further studies of non-halophytes are needed.

## Sources of Funding

Funding for this project was provided by the University of Manitoba (University Research Grant Program, Winnipeg, Canada) to S.R. and by The University of Winnipeg through a Major Research Grant (Winnipeg, Canada) to G.A.S.

## Controbutions by the Authors

S.W. was the student who conducted the experiment. G.A-S. and S.R. co-supervised the student and designed the experiment. J.M. and G.A-S. conducted the statistical analysis. J.M., G.A-S. and S.R. contributed equally to the writing of the manuscript.

## Conflict of Interest Statement

None declared.
